# An Amino Acid Substitution (L925V) Associated with Resistance to Pyrethroids in *Varroa destructor*

**DOI:** 10.1371/journal.pone.0082941

**Published:** 2013-12-18

**Authors:** Joel González-Cabrera, T. G. Emyr Davies, Linda M. Field, Peter J. Kennedy, Martin S. Williamson

**Affiliations:** Department of Biological Chemistry and Crop Protection, Rothamsted Research, Harpenden, Herts, United Kingdom; Ghent University, Belgium

## Abstract

The Varroa mite, *Varroa destructor*, is an important pest of honeybees and has played a prominent role in the decline in bee colony numbers over recent years. Although pyrethroids such as tau-fluvalinate and flumethrin can be highly effective in removing the mites from hives, their intensive use has led to many reports of resistance. To investigate the mechanism of resistance in UK Varroa samples, the transmembrane domain regions of the *V. destructor* voltage-gated sodium channel (the main target site for pyrethroids) were PCR amplified and sequenced from pyrethroid treated/untreated mites collected at several locations in Central/Southern England. A novel amino acid substitution, L925V, was identified that maps to a known hot spot for resistance within the domain IIS5 helix of the channel protein; a region that has also been proposed to form part of the pyrethroid binding site. Using a high throughput diagnostic assay capable of detecting the mutation in individual mites, the L925V substitution was found to correlate well with resistance, being present in all mites that had survived tau-fluvalinate treatment but in only 8 % of control, untreated samples. The potential for using this assay to detect and manage resistance in Varroa-infected hives is discussed.

## Introduction

The infestation of Western honey bees (*Apis mellifera*) by the ectoparasitic mite *Varroa destructor* has been an important factor contributing to the increasing losses of colonies reported particularly in many European countries [1]. The mites feed on the haemolymph of adult bees and their larvae, adversely affecting their health and making them more susceptible to Varroa-transmitted pathogens such as bee viruses [2]. Without an effective treatment programme, a Varroa infestation is likely to result in the decline and death of the entire hive within 2–3 years [3].

The control of *V. destructor* has relied heavily on chemical interventions, with certain synthetic pyrethroids (eg. tau-fluvalinate and flumethrin) among the most useful products for providing selective and effective control of the parasite without harming the bees on which they feed [1]. Unfortunately, widespread use of these compounds, as with most pesticides, has led to selection for resistance and reports of reduced efficacy of pyrethroid products against Varroa are now common place. Following initial reports in Europe during the mid 1990s [4], resistance has now been reported from nearly all parts of the world [4]–[12] and has resulted in a gradual decline in the use of pyrethroids and their replacement by less effective chemical treatments [13].

Pyrethroids act on the nervous system of arthropods where they modify the gating kinetics of the voltage-gated sodium channel (VGSC), a large transmembrane protein that generates the rising phase of action potentials in neurones and other excitable cells [14]. The disruption of gating results in erratic discharges and loss of co-ordinated neural signalling, eventually leading to paralysis and death [15]. Although most pyrethroids show high potency against both insects and mites, certain compounds with larger acid side groups show a marked selectivity for mites and ticks [16]. These acaricidal pyrethroids, for example tau-fluvalinate and flumethrin, are therefore useful in situations where insecticidal activity would be harmful, such as the control of Varroa mites in bee hives.

Resistance to pyrethroids has been recorded in a wide range of crop and disease pests and most commonly results from mutations in the VGSC gene that cause amino acid substitutions in the channel protein [17], [18]. These substitutions tend to group into ‘hot-spots’ for resistance and many are located within the transmembrane segments of domains II and III that recent modelling studies have identified as forming a possible binding site for pyrethroids close to the channel pore [19]. The most common mutation is leucine 1014 to phenylalanine (L1014F) and is located in the segment IIS6 of domain II. This mutation is generally associated with moderate (10–30 fold) resistance to pyrethroids and has been termed the kdr (knockdown resistance) mutation [20]. A number of mutations have also been identified that are associated with stronger (super-kdr) resistance and these include substitutions at methionine 918 (IIS4-S5 linker), leucine 925, threonine 929, leucine 932 (IIS5 helix) and phenylalanines 1534, 1538 (IIIS6 helix) (reviewed in [17], [18]).

The molecular basis underlying tau-fluvalinate/flumethrin resistance in *V. destructor* is currently not known. There is evidence to suggest that target site modification is the most likely mechanism here too [21] and, although previous studies in the USA and Korea have compared VGSC sequences from susceptible and resistance mite populations [5], [11], the amino acid substitutions that were identified did not correspond to any of those found in known hot-spots for resistance. Here, we report VGSC sequencing of Apistan® (tau-fluvalinate)-treated and untreated mite samples from different locations in Central/Southern England. We identify a novel point mutation that maps to a hot-spot for resistance within the proposed VGSC binding site for pyrethroids and shows an excellent correlation with resistance in the tau-fluvalinate treated samples.

## Materials and Methods

Ethical statement. The samples (adult mites) used in this study were voluntarily provided by individual beekeepers knowing that the results would be used for a scientific publication.

*Varroa destructor*. Samples (adult mites) used for DNA sequencing studies were mostly collected from apiaries where the beekeepers had reported a reduced efficacy of Apistan® (tau-fluvalinate) to control *V. destructor* infestations. Samples C and D were collected in August 2009 from hives located in Harpenden (Hertfordshire, UK) three days after the 6-week treatment with Apistan® had been completed. Samples V8 and V10 were collected in 2012 from hives located near Hitchin (V8: Henlow and V10: Shillington, both Bedfordshire, UK), 5½ weeks after a 7-week treatment with Apistan® had been completed and the lack of efficacy had been implied by continued high Varroa counts. A control sample (DL) was collected from a Harpenden hive that had not been treated with Apistan® for at least 3 years. All mites were collected alive; those from Hertfordshire from assessment boards below mesh hive floors using a fine paint brush; those from Bedfordshire using an icing sugar roll method [22]; all were immediately frozen in dry ice and then stored at –80°C.

Samples tested with the TaqMan diagnostic assay were collected from several sites in Central/Southern England (see Table 1). In this case the mites (both dead and alive) were separated from floor debris using a fine paint brush and stored at –20°C.

**Table 1:** Sample locations and genotyping results using the L925V TaqMan diagnostic assay on individual Varroa mites collected from several sites in Central/Southern England. Genotype SS  =  L925 wild-type, SR  =  L925V heterozygote, RR  =  V925 resistant homozygote.

Location	Treatment status^1^	Genotype			Total
		SS	SR	RR	
Harpenden	Non-treated	119	9	7	143
Bishop Stortford		32	0	0	32
St. Albans		32	0	0	32
Peterborough		23	2	7	32
Henlow	Apistan® Treated	0	0	8	8
Shillington		0	1	31	32

® (tau-fluvalinate) treated mites were collected one month after the end of the treatment.^1^ Non-treated mites were collected from hives that had not been treated with pyrethroids for at least one year. Apistan

**Figure 1:**
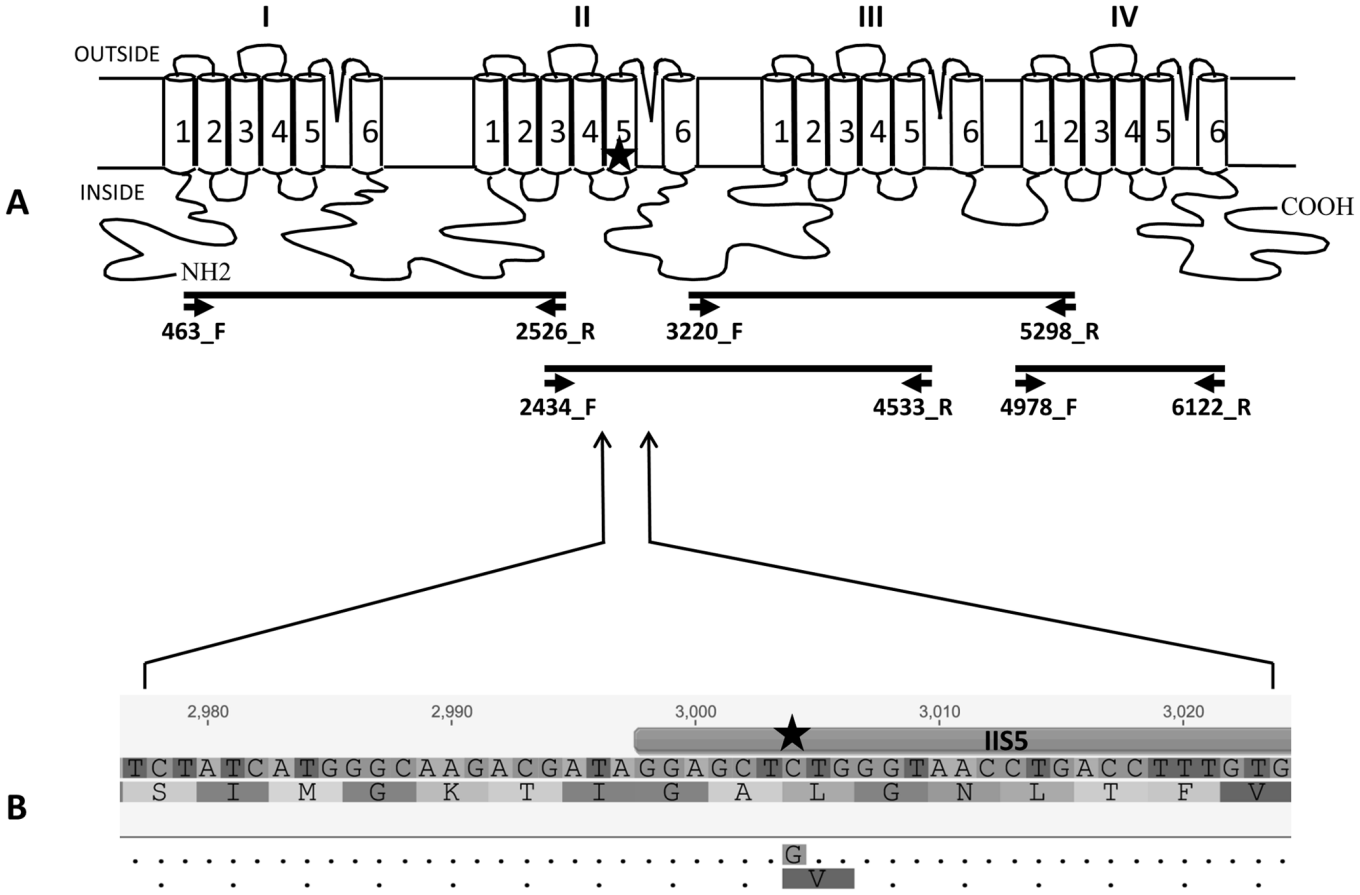
Schematic diagram of the *Varroa destructor* sodium channel gene and position of the mutation L925V. **A:** Diagram of the sodium channel protein showing the four main domains (I–IV) and proposed folding of the membrane segments (S1–S6) within each domain. **B:** Nucleotide and amino acid sequences of the IIS4–IIS5 linker & IIS5 helix flanking the L925V mutation. Identical nucleotide and amino acid residues in the ‘resistant’ sequence are shown as dots. The star indicates the position of the mutation.

Analysis of sequences encoding Domains I to IV. The mites were ground in liquid nitrogen (pools of 4 to 16 mites depending on availability) and total RNA was extracted using the Isolate RNA mini kit (Bioline) according to the manufacturer’s recommendations. cDNA was reverse transcribed from the RNA (0.5–1 µg) using Maxima H minus First Strand cDNA synthesis kit (Thermo Scientific), oligo dT_18_ (250 ng) and the specific primer 4256_R_Vd (10 ng) (Table 2). First strand cDNA was used as a template for PCR amplification of four overlapping fragments covering domains I to IV of the *V. destructor* VGSC (Fig. 1). Each fragment was amplified by a two-step nested PCRs [23] using the primer combinations shown in Table 3 where PCR 1 refers to the primary amplification and PCR 2 the secondary amplification. For each PCR, 1 µl of cDNA (or PCR 1) was mixed with 100 ng of each primer, 12.5 µl of DreamTaq Green PCR Master Mix (2×) (Thermo Scientific) and water to a final volume of 25 µl. Cycling conditions were: 94°C for 2 min followed by 35 cycles of 94°C for 45 s, 60°C for 45 s and 72°C for the appropriate extension time (see Table 3), and final extension at 72°C for 5 min. The PCR fragments were ethanol precipitated and direct sequenced (Eurofins MWG Operon, Germany) using the following set of primers (Tables 2 and 3): Fragment I: 653_F_Vd, 1356_F_Vd, 2043_F_Vd, 753_R_Vd, 1457_R_Vd and 2150_R_Vd; Fragment II: 2751_F_Vd, 3448_F_Vd, 2843_R_Vd, 3317_R_Vd, 3556_R_Vd and 4256_R_Vd; Fragment III: 3448_F_Vd, 4146_F_Vd, 4846_F_Vd, 3556_R_Vd, 4256_R_Vd, 4533_R_Vd and 4949_R_Vd; Fragment IV: 4978_F_Vd, 5560_F_Vd, 5650_R_Vd and 6122_R_Vd. The sequences were analysed using Geneious (Version 6.1 created by Biomatters. Available from http://www.geneious.com/).

**Table 2:** Oligonucleotides used to sequence *V. destructor* VGSC PCR fragments.

Primer	Sequence 5'-3'
653_F_Vd	CCGAAACAATATTCACGACG
1356_F_Vd	CAATCTGATTCTCGCCATTG
2043_F_Vd	CGTAGACGCTCAGGAACACC
2751_F_Vd	TTTCTTCACCGCTACCTTCG
3448_F_Vd	GGTAAACAGCGCAACCAGAT
4146_F_Vd	CGCAGAAGGAAAAGAAAACG
4846_F_Vd	GGCAGATTCTACCACTGCGT
5560_F_Vd	CTTTCGATCCTTGGCACAGT
753_R_Vd	CCATGGATCCCCAAGATATG
1457_R_Vd	AATCGCATAGCCTCTTCCAA
2150_R_Vd	TGCTGCAAATTGGAGTAGAGA
2843_R_Vd	TCAAATATATTCCACCCTTCTTT
3317_R_Vd	AACGAAGAGAGCAACAGGGC
3556_R_Vd	GCGTGGGGCTATCCTTCTTA
4256_R_Vd	TCAGGGATGATGAGGTCAGA
4949_R_Vd	GGGTTTTTCCAGGTAAAGTTGTT
5650_R_Vd	CAACCTTAACGACGCGTACA

*V. destructor* VGSC as template (Genbank accession number: AY259834 [34]). Primer nomenclature is as follows: the numbering indicates the template position corresponding to the 5′ end of the forward (F) or Reverse (R) primer, Vd is an abbreviation for *Varroa destructor*. All primers were designed using Geneious (Biomatters Ltd) using the complete coding sequence of the

**Table 3:** Oligonucleotides used to PCR amplify overlapping fragments of the *V. destructor* VGSC.

Fragment	Nested PCR	Primer	Sequence 5'-3'	Extension time (s)	Product Size (bp)
I	1	452_F_Vd	CGTTCATGGTGATCAGCAAGGGCAA	120	2099
		2550_R_Vd	ACAGCCAGCGAGACACTTTTCCT		
	2	463_F_Vd	ATCAGCAAGGGCAAAGACAT	120	2064
		2526_R_Vd	CCATTTGGCTTCGACATCTT		
II	1	2420_F_Vd	CAGCTGGCCGCCAAAGCAAG	135	2182
		4601_R_Vd	AAGTCGAGCCAACACCACGC		
	2	2434_F_Vd	AGCAAGGCCAGCGAGAGAGT	135	2100
		4533_R_Vd	TTCGGAGAAGAAGATTACGGTGAACG		
III	1	3170_F_Vd	GGGTGCTATGCGGCGAGTGG	135	2227
		5396_R_Vd	GCCATCACTGTCATGTTGAGCACG		
	2	3220_F_Vd	TCGGGCTGGCCCTGTATCCC	120	2079
		5298_R_Vd	TGGCCGTGGAATAGCCTTTGCG		
IV	1	4967_F_Vd	ACGTGCTCAATGCCTATTTGGCT	75	1200
		6166_R_Vd	TGGGGTCGAACTGCTGCCA		
	2	4978_F_Vd	GCCTATTTGGCTTTGTTCCA	75	1145
		6122_R_Vd	TCGTCCGTTAGACCTTCCTG		

*V. destructor* VGSC as template (Genbank accession number: AY259834 [34]). Primer nomenclature is as defined in Table 2. All primers were designed using Geneious (Biomatters Ltd) using the complete coding sequence of the

Unless otherwise stated, the numbering of amino acid residues in the VGSC of *V. destructor* matches that of the *Musca domestica* sodium channel (EMBL accession no X96668).

TaqMan diagnostic assays. Sequences of the *V. destructor* VGSC obtained in this study were used to design primers (flanking the L925V mutation site) and two minor groove-binding probes (MGB) (Life technologies) using the Primer Express™ Software v.2.0 (Life Technologies). Forward Vd_L925V_F (5′-CCAAGTCATGGCCAACGTT-3′) and reverse Vd_L925V_R (5′-AAGATGATAATTCCCAACACAAAGG-3′) primers were standard oligonucleotides with no modification. The probe Vd_L925V_V (5′-TTACCCAGAGCTCC-3′) was labelled with the fluorescent dye VIC® at the 5′ end for the detection of the wild-type allele, and the probe Vd_L925V_M (5′-TTACCCACAGCTCCT-3′) was labelled with the fluorescent dye 6FAM™ for detection of the L925V mutation. Each probe also had a 3’ non-fluorescent quencher and a minor groove binder at the 3’ end. This minor groove binder increases the Tm between matched and mismatched probes providing more accurate allele discrimination [24].

Genomic DNA was extracted from adult mites essentially as described by Stanton et al. (1998) [25] but with several modifications. Briefly, the mites were placed in a 96-well plate (one mite per well) and incubated at 99°C for 3 min in 20 µl of 0.25 M NaOH. Then they were ground with a plastic homogeniser and the solution was neutralised adding 10 µl of 0.25 M HCl, 5 µl of 0.5 M Tris-HCl and 5 µl of 2 % Triton X-100. The plate was further incubated as above and then spun at 3200 ×*g* for 5 min. The supernatant containing the genomic DNA was stored at –20°C.

TaqMan assays contained 1.5 µl genomic DNA, 7.5 µl of 2x SensiFAST™ probe Hi-Rox Mix (Bioline), 0.9 µM each primer and 0.2 µM each probe in a total reaction volume of 15 µl. Assays were run on an ABI 7900HT Real-Time PCR system (Applied Biosystems) using the temperature cycling conditions: 10 min at 95°C followed by 40 cycles of 95°C for 15 s and 60°C for 45 s. The increase in VIC® and 6FAM™ fluorescence was monitored in real time by acquiring each cycle on the yellow channel (530 nm excitation and 555 nm emission) and green channel (470 nm excitation and 510 emission) of the ABI 7900HT, respectively.

## Results

Total RNA was isolated from pools of *V. destructor* adults collected from bee hives either treated (samples C, D, V8 and V10) or non-treated (sample DL) with Apistan® (tau-fluvalinate). The RNA was reverse transcribed into cDNA to amplify the four fragments encompassing domains I, II, III and IV of the *V. destructor* VGSC (Fig. 1, Table 3). These fragments covered 85 % of the total coding sequence for this gene (5,659 bp out of 6,648 bp). Comparison of the sequences identified a single point mutation (C to G at nucleotide 3004) that differentiated between treated and non-treated samples (GenBank accession numbers: KF771990-KF771994 for samples DL, C, D, V8 and V10, respectively). This mutation was only seen in the treated samples C, D, V8 and V10 and results in a leucine (CTG) to valine (GTG) substitution at position 925 of the VGSC protein. A second polymorphism was also observed that differentiated between samples collected in different locations. Hence, samples DL, C and D (collected in Harpenden) showed a G to C polymorphism at base 3952 compared to samples V8 and V10 (collected in Henlow and Shillington, respectively) causing an arginine (CGT) to glycine (GGT) substitution at channel residue 1318 (Varroa numbering as this region is not conserved in the housefly VGSC). A further polymorphism (G to A at base 5997 causing methionine (ATG) and isoleucine (ATA) at position 1823) appeared as a ‘mixed’ allele in all of the samples. These substitutions seemed unlikely to be involved in pyrethroid resistance (see discussion) so were not explored further.

**Figure 2:**
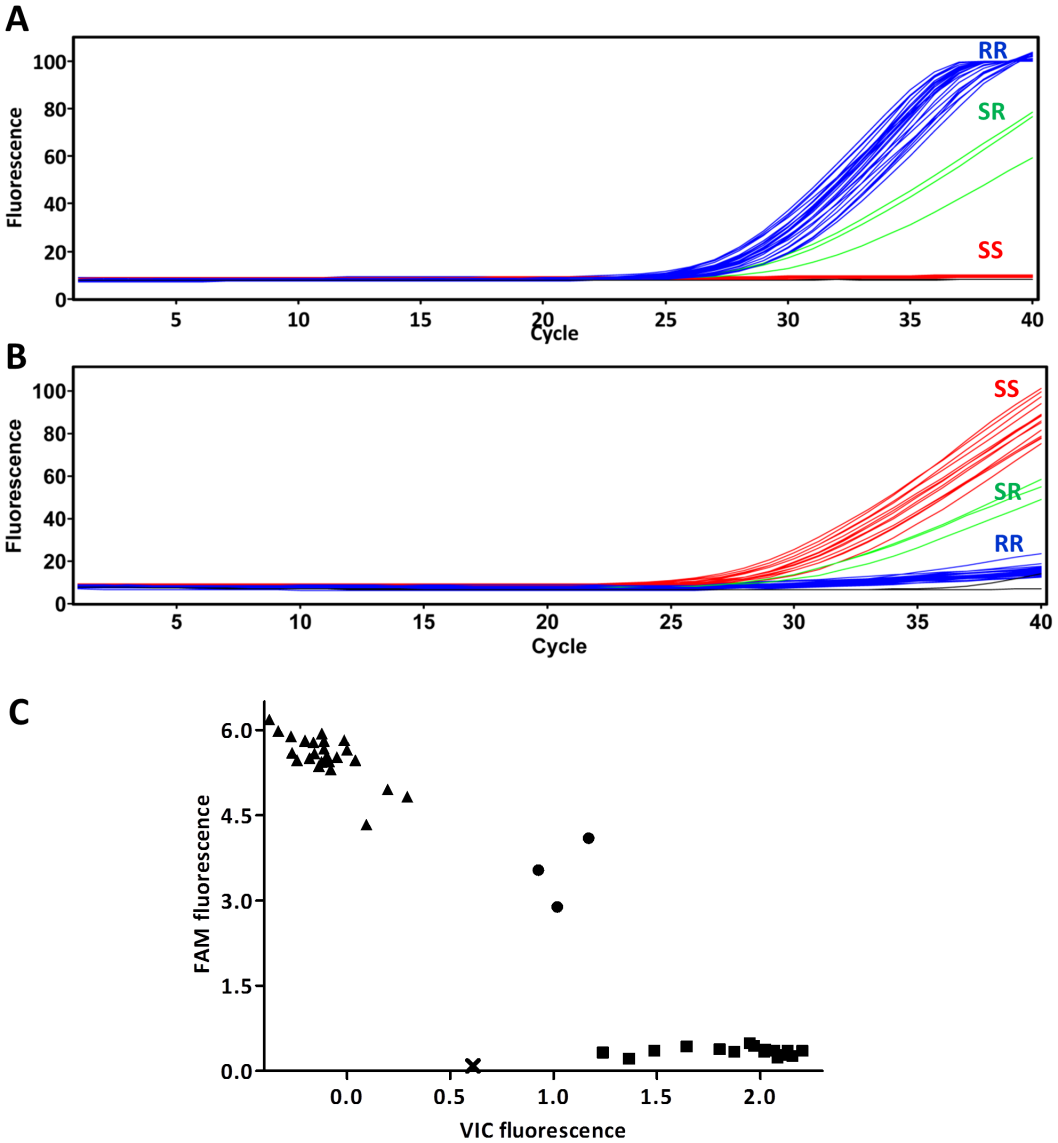
Real-time TaqMan detection of the L925V mutation in *Varroa destructor*. **A:** Cycling of 6FAM™-labelled probe specific for the V925 (resistant) allele. **B:** Cycling of the VIC®-labelled probe specific for the L925 (wild-type) allele. RR  =  resistant homozygote, SR  =  heterozygote, SS  =  wild-type. **C:** Scatter plot analysis of corrected 6FAM™ (V925 allele) and VIC® (L925 allele) fluorescence data showing clear separation of the RR homozygotes (triangles), RS heterozygotes (circles) and SS homozygotes (squares).

To confirm the association of the L925V mutation with mites from pyrethroid-treated colonies, a TaqMan allelic discrimination assay was developed to enable rapid genotyping of individual mites from a number of other sites in Central/Southern England (Fig. 2, Table 1). This is a real-time PCR assay that uses two fluorescent labelled probes to discriminate between wild-type (L925) and mutated (V925) alleles. The first probe, selective for the L925 allele, is labelled with VIC® while the other probe, selective for V925, is labelled with 6FAM™. An increase in VIC® fluorescence therefore indicates the presence of the wild-type allele, while an increase in 6FAM™ fluorescence indicates the presence of the mutant allele. An intermediate increase in the fluorescence of both dyes indicates that the mite is heterozygous for the mutation (Fig. 2A, B).

The genotyping of 279 individual mites collected from treated as well as from non-treated hives showed a strong correlation between the treatment with tau-fluvalinate and the presence of the mutation V925 (Table 1). All of the 40 mites collected from treated hives carried the V925 mutation, with 39 scoring as homozygotes and one as a heterozygote. In contrast, the 239 mites collected from non-treated hives showed a more heterogeneous pattern with 206 homozygous for the wild-type L925 and just 25 carrying the V925 mutation (11 as heterozygotes and 14 as V925 homozygotes). This represents a V925 (resistant) allele frequency below 10 % in the untreated samples.

## Discussion

The evolution of resistance to pesticides in field populations of insects and mites continues to be a major threat to the long-term success of pest control programs based on conventional pesticides. In the case of *V. destructor*, the control of the parasite has relied heavily on a small number of active ingredients with the synthetic pyrethroids (tau-fluvalinate and flumethrin) among the most effective and widely used [1] and so it is perhaps not surprising that reports of resistance to these compounds are now common [4]–[9], [11]. Although this resistance dates back to the 1990’s, there is at present no clear description of the exact mechanism(s) involved. Studies in other insect and mite species have identified a relatively small number of mutations in the VGSC target that correlate with resistance to pyrethroids [17], [18], however previous work on resistant populations of V. destructor from the USA and Korea did not discover any of the ‘common’ resistance mutations, and have so far resulted in several amino acid substitutions in regions that are not generally associated with resistance [5], [11]. Of these, only one (L1596P) has been tested for functionality (by expression in *Xenopus* oocytes) and shown to confer low (5-fold) reduced sensitivity to pyrethroids [26].

In the present study, we identify a single amino acid substitution (leucine 925 to valine, L925V) in the VGSC of *V destructor* samples from the UK that is located at a known resistance ‘hot-spot’. This mutation was found in all of the mites that survived Apistan® (tau-fluvalinate) treatment. In contrast, the frequency of the mutation in control mites from untreated colonies was less than 10 %. The Apistan® strips contain 10 % tau-fluvalinate which is normally sufficient to eliminate 98 % of susceptible mites [27], indicating that the presence of the mutation is tightly linked to the resistant phenotype. Leucine 925 is part of the domain IIS5 transmembrane helix and is positioned between two other residues that are well known mutation sites for resistance, methionine 918 and threonine 929. Although the exact mutation reported here, L925V, has not been observed in other species, the closely related substitution to isoleucine, L925I, has been correlated with resistance in at least four other species*; Bemisia tabaci, Cimex lectularius, Rhipicephalus microplus and Trialeurodes vaporariorum* [28]–[30]. Mutations at all these residues are frequently associated with strong (so called super-kdr) levels of resistance to pyrethroids and the functionality of mutations at these residues has been confirmed by expression and electrophysiological analysis of mutated channels in *Xenopus* oocytes [17], [18], [31]. We infer a similar role for L925V based on its chemical similarity to L925I and also note that multiple mutations at M918 (to threonine, leucine, valine) and T929 (to isoleucine, valine, cysteine) have all been reported in other pest species [17], [18]. Furthermore, a direct role for L925 in the binding of pyrethroids at insect and acari sodium channels has been proposed from molecular modelling studies. By constructing a homology model of the insect sodium channel based on the crystal structure of a potassium channel from rat brain Kv1.2 [32], O’Reilly et al (2006) identified a hydrophobic binding pocket comprising residues of the IIS4/S5 linker, IIS5 and IIIS6 helices that could accommodate a range of pyrethroid structures. L925 (like M918 and T929) faces into this pocket and provides side chain interactions that are suggested to stabilise binding of pyrethroids within this pocket [19]. This would then explain why any changes in the side chain conformation of these residues could de-stabilise binding and result in resistance. We believe that the fact that our analysis found that only mites containing this mutation survived Apistan® treatment, as well as the location of this mutation within the proposed pyrethroid binding site, provides compelling evidence that L925V is likely to be the primary cause of resistance in the *V destructor* populations tested here.

We note that two other amino acid substitutions were identified in our sequencing studies, R1318G (Varroa numbering) and M1823I. A role for either of these substitutions in conferring resistance seems less likely because neither showed the same correlation with pyrethroid treatment survival that was observed for L925V. The R1318G substitution shows a clear geographical separation with all samples from one location (DL, C, D) containing R1318, while samples from other locations (V8, V10) contained G1318. Furthermore, this residue is located within the long intracellular linker between channel domains II and III, a highly variable region between species with no known role in pyrethroid mode of action or resistance. M1823I shows a more random distribution within our samples; it was present in all sequenced samples but spread evenly between those surviving or killed by the pyrethroid treatment. This residue is located in domain IVS6 and is likewise a region not previously associated with resistance mutations in other species [17], [18].

The discovery of a single point mutation, L925V, within the *V. destructor* VGSC provides new opportunities for the management of resistance in this important parasite. Our analysis shows that while this mutation may dominate in hives that have undergone recent pyrethroid treatment, the frequency is low in the Varroa populations of hives that have not been subjected to recent treatments (Table 1). This indicates that despite the long history of resistance problems dating back almost 20 years, the mutation is not universal and may not survive well in local populations in the absence of pyrethroid selection. This is a common feature in resistance management and is often associated with a reduced fitness of resistant individuals in the absence of selection [33]. If indeed there is a high level of variability in the distribution and frequency of the mutation in local hive populations there could well be scope for re-using pyrethroids in areas where the mutation is not currently present or at very low frequency and we believe this would be welcomed by many beekeepers since pyrethroid products, when working efficiently, are one of the safer and more effective ways of removing the mites from a hive. In order to maintain manageable levels of the mutation once the selection pressure is re-imposed, pyrethroids would of course need to be used in strict rotation with other chemicals and in combination with other approaches as part of an effective integrated management system. In this context, the development of the high throughput TaqMan assay for detecting the mutation would be particularly useful. The advantage of DNA-based assays for mutation detection and resistance monitoring are their relatively low cost, speed, and ability to test poor quality and even dead samples. Indeed, we have found that the assay described here is extremely robust and capable of accurately genotyping individual dead mites collected from hives and stored at ambient temperatures over several days. This in turn makes it feasible to assess the status of individual hives for the presence and/or frequency of the mutation before deciding whether treatment with pyrethroids is likely to be successful. Due to the likelihood of the mutation being re-selected to higher levels following treatment, as was the case with the Apistan®-treated hives tested here, careful monitoring of the distribution and frequency of the mutation in local Varroa populations using the diagnostic assay will be necessary. Nevertheless, it should be possible to develop a pro-active monitoring programme using a rotation of different products (including pyrethroids) aimed at managing resistance and maintaining a more effective control of this highly damaging pest.
